# Towards More Efficient Security Inspection via Deep Learning: A Task-Driven X-ray Image Cropping Scheme

**DOI:** 10.3390/mi13040565

**Published:** 2022-03-31

**Authors:** Hong Duc Nguyen, Rizhao Cai, Heng Zhao, Alex C. Kot, Bihan Wen

**Affiliations:** School of Electrical & Electronic Engineering, Nanyang Technological University, Singapore 639798, Singapore; e200215@e.ntu.edu.sg (H.D.N.); rzcai@ntu.edu.sg (R.C.); zhaoheng@ntu.edu.sg (H.Z.); eackot@ntu.edu.sg (A.C.K.)

**Keywords:** X-ray imaging, objective detection, image cropping, deep learning, features extraction

## Abstract

X-ray imaging machines are widely used in border control checkpoints or public transportation, for luggage scanning and inspection. Recent advances in deep learning enabled automatic object detection of X-ray imaging results to largely reduce labor costs. Compared to tasks on natural images, object detection for X-ray inspection are typically more challenging, due to the varied sizes and aspect ratios of X-ray images, random locations of the small target objects within the redundant background region, etc. In practice, we show that directly applying off-the-shelf deep learning-based detection algorithms for X-ray imagery can be highly time-consuming and ineffective. To this end, we propose a Task-Driven Cropping scheme, dubbed TDC, for improving the deep image detection algorithms towards efficient and effective luggage inspection via X-ray images. Instead of processing the whole X-ray images for object detection, we propose a two-stage strategy, which first adaptively crops X-ray images and only preserves the task-related regions, i.e., the luggage regions for security inspection. A task-specific deep feature extractor is used to rapidly identify the importance of each X-ray image pixel. Only the regions that are useful and related to the detection tasks are kept and passed to the follow-up deep detector. The varied-scale X-ray images are thus reduced to the same size and aspect ratio, which enables a more efficient deep detection pipeline. Besides, to benchmark the effectiveness of X-ray image detection algorithms, we propose a novel dataset for X-ray image detection, dubbed SIXray-D, based on the popular SIXray dataset. In SIXray-D, we provide the complete and more accurate annotations of both object classes and bounding boxes, which enables model training for supervised X-ray detection methods. Our results show that our proposed TDC algorithm can effectively boost popular detection algorithms, by achieving better detection mAPs or reducing the run time.

## 1. Introduction

X-ray screening is a commonly-used security measure at airports, border checkpoints and public transportation due to the merits such as real-time imaging and non-invasiveness. The investigation of X-ray images was typically done by human screeners, while such a manual process is expensive, inefficient, tiring, and can be affected by factors such as mental exhaustion and workplace conditions [1,2]. Additionally, the different view angles of clutter and overlapped objects in security X-ray images will further increase the risk of missing prohibited items [3,4]. Owing to these reasons, it is desired to automate the X-ray screening process using advanced object detection algorithms.

**Figure 1:**
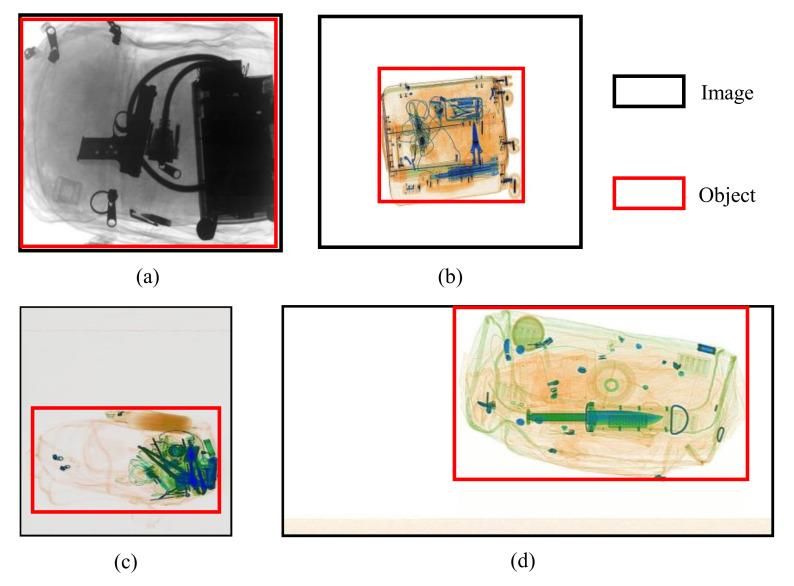
Examples of security X-ray images from popular public datasets: (**a**) GDXray [14], (**b**) OPIXray [13], (**c**,**d**) SIXray [17].

While classic methods [5,6,7,8] exploited image processing and model-based optimization for X-ray object detection, recently proposed deep learning algorithms [9,10,11,12,13] have proved to be a better method with higher detection accuracy. Along with an increase in the number of available detection algorithms, more security X-ray image datasets [13,14,15,16] are published and made publicly available from 2016 onward, which enable more deep learning methods to solve the X-ray image detection or classification problems. Although providing valuable auto-detection benchmarks, these datasets all share the same common trait: the images are manually cleaned and processed, i.e., the objects of interest are manually amplified and centered to fit the viewport. However, in the realistic situations, the scanned items do not always fit the viewport and may appear very small compared to the redundant background. Figure 1 show some examples of the manually process X-ray images from the GDXray [14] and OPIXray [13] datasets, compared to the realistic images from Sixray [17]. Besides, to run the trained detection models, X-ray images are typically required to be resized to square, but such process may distort the original resolution and aspect ratios of the relevant objects. Research has also pointed out that the smaller sizes of the input image can decrease the detector’s accuracy [18,19,20]. The redundant spaces also contribute nothing to the detection’s ability but more processing time, as large size input images tend to increase the inference time of the model, so a method of removing such spaces is much needed. Conventional cropping techniques such as center cropping or edge cropping might not perform well on security X-ray images due to the variety in size and objects’ location along with X-ray artifacts [21,22]. Alternatively, deep learning-based cropping or retargeting techniques [23,24,25,26] using feature extraction mainly focus on the aesthetic aspect of images. They focus on improving the visual quality and often neglect the inference time of the model and the effect of cropping on detection time and accuracy.

In this work, we propose Task-Driven image Cropping (TDC)—a novel and efficient scheme of cropping security X-ray images using activation output of convolutional layers of the detection network to simultaneously achieve two objectives, i.e., reduction of run time and improvement of detection accuracy. By utilizing the change in the energy of the feature maps extracted from the network backbone, we can efficiently crop the unwanted background and preserve regions of interest. To test the cropping performance of TDC for real-life classification and detection problems, we select SIXray [17] because it provides extra challenges with multiple viewpoints, complex backgrounds, and overlapping objects. However, only the test SIXray dataset has bounding box-level annotation and some samples are wrongly annotated as negative (without prohibited items) in the dataset. To provide better annotations, we propose a novel X-ray image detection benchmark, named SIXray-D, which is a fully annotated detection dataset with more positive images and objects.

Our contribution can be summarized as follows:

1. We propose SIXray-D, an improved dataset based on the popular SIXray [17] as a fully annotated dataset for contraband items detection. SIXray-D provides a comprehensive detection benchmark, which can be used to evaluate and improve the effectiveness of deep X-ray detection networks.
2. We propose TDC, a task-driven X-ray image cropping pipeline to efficiently remove redundant background and preserve the task-related objects by utilizing the features extracted from the network’s backbone.
3. We conduct experiments to evaluate several state-of-the-art single-stage detectors on the proposed SIXray-D. We show that TDC can effectively improve the detection methods such as RFB-Net, by achieving better mAPs or reducing the inference time.

The remainder of this paper is organized as follows. Section 2 briefly introduces the background and related works. Section 3 presents our proposed SIXray-D dataset for X-ray image detection tasks. Section 4 describes the details of the proposed TDC scheme and how it improves the X-ray image detection tasks. The experimental results are presented in Section 5, and Section 6 provides several concluding remarks.

## 2. Related Works

**X-ray security inspection task.** X-ray screening is a universal security measure at border checkpoints, airports, and public transportation due to the thoroughly real-time imaging and non-invasiveness. The procedure can be conducted on both passengers and their luggage to identify any prohibited and potentially dangerous items carried through the border checkpoints or stations. The inspection of X-ray images is mostly done by security personnel, and human factors such as physical well-being, mental health, and work satisfaction could affect the process and lead to errors in detecting contraband items. According to a survey on airport security professionals in 18 Brazilian airports [2], 61% of the professionals admit that they have committed an error during security inspection and try to correct it. The main factors contributing the most to the errors are the tiring, repetitive and monotonous jobs, the neglect of following work procedures, and the complacency on the jobs. Furthermore, training the human operators for threat detection is expensive and requires a great amount of effort, hence automated X-ray screening process is desired using object detection algorithms.

**X-ray security dataset.** There are several public datasets in the X-ray security field such as the GDXray dataset [14] and OPIXray dataset [13]. Each dataset has a different way of labeling images, and the number of classes is also varied. These datasets contain non-complex X-ray images with no redundant background, and there is usually only one object of interest per image. Moreover, the proportion between positive and negative samples does not reflect realistic scenarios, thus making such datasets non-ideal for security inspecting applications. HiXray [27] is another security X-ray inspection dataset with high-quality images, multiple objects of interest per image, and object occlusion. However, it focuses on airport cabin baggage as the classes consist of phone charger, water bottle, mobile phone, tablet, and laptop, which are usually scanned separately with airplane cabin baggage. On the other hand, these items are allowed in checked-in baggage and general security X-ray inspection such as subway station or land border checkpoint. Furthermore, the images are pre-processed, and the luggage is situated at the center of the viewport, thus making the dataset less realistic. Meanwhile, SIXray [17] provides a complex dataset with extra challenges such as overlapped objects, various image sizes, and difficult background content. It proves to be the largest security X-ray dataset up to date with over 1 million images, including both positive and negative samples. However, the manual annotations for SIXray are done coarsely with only the test set annotated with bounding boxes, hence more detailed annotations on the whole dataset can lead to improvement in the accuracy of the detection model.

**Single-stage detectors.** Top-performing image detectors are usually two-stage detectors using deep convolutional neural networks (CNN) backbones such as Inception [28], Mask-RCNN [29], or ResNet [30] with the trade-off of high computational complexity and slow runtime. Alternatively, several one-stage detectors provide real-time speed and performance comparable to these two-stage detectors, namely RetinaNet [31], SSD [32]. RetinaNet utilizes feature pyramid networks [33], and couples with the ResNet [30] backbone network for feature extraction. It also introduces FocalLoss to handle the class imbalance problem of a single-stage detector to provide a good detection performance. SSD introduces the application of dividing the image into a grid with the pre-defined anchor boxes in each grid cell. The anchor boxes have different sizes and aspect ratios, and they are responsible for matching the objects of interest during the training and detecting process. Liu et al. [34] proposed a lightweight detector consisting of Receptive Field Block (RFB) modules inspired from Receptive Fields (RFs) in human eyes to make features more distinguishable on top of the SSD network. Besides, with the recent work of YOLOv5 [35] and image transformer for one-shot detector [36], the performance of single-stage detectors could surpass their double-stage detector counterparts in some experiments. X-ray image detection at a security checkpoint requires fast inference time, thus by using single-stage detectors on the task we can achieve good accuracy and relatively real-time detection speed.

**Image retargeting/cropping methods.** Image cropping and retargeting are popular methods to remove the redundant area of an image. The main goal of image retargeting [25,37,38,39] is improving the aesthetic of the image by warping but with a risk of warping objects of interest and generating artifacts. Alternatively, image cropping is much simpler, as it only selects an area that contains saliency regions. The SIXray dataset provides a challenge to auto image cropping methods such as center cropping due to the different locations of objects and variety of image sizes. Additionally, X-ray artifacts [21] such as vertical or horizontal bars prevent cropping using conventional methods such as edge detection [40] to crop the interest region. Furthermore, image retargeting usually requires a target aspect ratio [25,38,39], which is hard to determine due to the dataset’s characteristics.

**Object detection in security X-ray inspection.** The methods for automated object recognition in X-ray can be categorized into conventional image analysis, machine learning-based approach, and deep learning-based approach. Classic methods range from fusion, de-noising, and enhancement of dual-energy X-ray images [6] to Threat Image Projection (TIP) [16] for enhancing X-ray detection performance. Such methods exploit image processing and threshold-based optimization to improve the operators’ performance and alertness. Before the rise in the number of deep learning-based algorithms, the bag of visual words (BoVW) was the popular machine learning method for both object classification [41,42] and object detection [43,44]. Besides BoVW, some other common approaches [45,46] based on feature descriptors and k-NN classifier [47] were used for multi-view X-ray images. Recently, with the introduction of deep learning algorithms, object detection methods based on both single-stage detectors [9,11,48] and double-stage detectors [48,49,50] prove to be the better choice for automated X-ray image inspection. Furthermore, pixel-level analysis [51] can be conducted to enhancing the performance of the deep learning detector for large-scale X-ray security images. They achieve high accuracy and reasonable inference time on different X-ray image datasets such as DBF3 [52], GDXray [14], and SIXray [17] datasets.

## 3. SIXray-D Dataset

GDXray [14] and OPIXray [13] are popular public security X-ray datasets serving the detection task, and the key attributes of these datasets can be summarized in Table 1. In GDXray and OPIXray datasets, images are manually cleaned, and redundant background and noise are removed as shown in Figure 1. The GDXray only contains simple settings and non-clutter baggage with one contraband item per image, hence the dataset does not align with the realistic situation where objects of interest appear together with other items and are hard to be detected. On another hand, the image in OPIXray has object occlusion, but it also has only one prohibited object per baggage. Besides, the dataset only consists of knives and scissors classes, thus excluding some contraband classes such as guns or wrenches. Furthermore, both GDXray and OPIXray have the trouble of retrieving the datasets from the sources.

Due to the aforementioned limitations, we choose to prepare our new benchmark using SIXray [17] as our data baseline, which provides more complex X-ray images, multiple contraband items per image, and easier accessibility. The original SIXray dataset has 8,929 positive images with contraband items and 1,050,302 negative images. The ratio between positive and negative samples of SIXray is around 1:1000, and it reflects the realistic frequency of appearance of prohibited items. Though SIXray intends to provide 6 object classes, images containing hammers were not available for download. Thus, in the proposed SIXray-D dataset, we proceeded with only 5 item classes, namely Pliers, Scissors, Gun, Knife, and Wrench. More importantly, the SIXray is designed for the classification task, hence originally only 1200 positive images were annotated at the bounding box level in the testing set. In practice, training a detection deep model with only a few annotated images usually leads to overfitting, e.g., the detection mAP for the original SIXray is only 65.62 [17] when using DenseNet [53].

**Figure 2:**
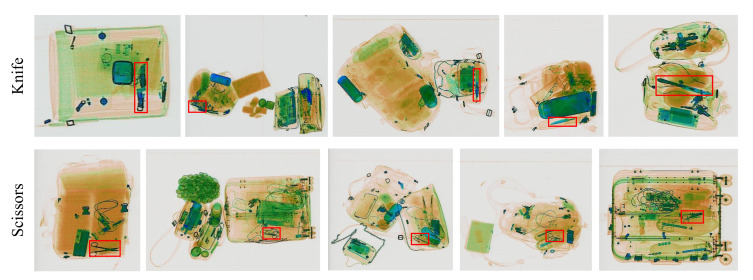
False-negative images from the SIXray dataset [17]. The red bounding boxes that are newly annotated by SIXray-D indicate the contraband items from the classes Knife and Scissors.

**Figure 3:**
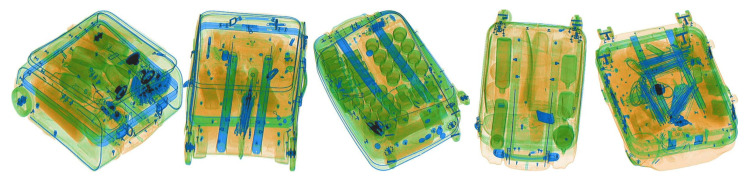
Negative images that are marked as positive by the detection network.

To this end, we propose a new X-ray object detection benchmark by utilizing the image data from SIXray [17] with more comprehensive annotation for detection, named SIXray-D. There are 8823 positive images in total that are publicly available from SIXray and around 1 million negative images. We train the RFB network for object detection using the positive images. Then, we apply the network to conduct detection on the negative images. The network detects some contraband items in these negative images (inspected set). We manually inspect the inspected set and we find that there are two categories. The first one is images that contain contraband items, but are the false-negative image of the SIXray dataset (wrongly annotated as negative). Such false-negative samples usually have a large area of redundant background with multiple overlapped objects, and the contraband items are small compared to the other components. Figure 2 illustrates some of the Scissors and Knife images that are false-negative. The second category does not contain contraband images but is marked as positive by our network. The second catagory is the false-positive images to the detector, and Figure 3 illustrates such images. In the proposed SIXray-D dataset, we complete the annotation for the first category images and add them to the positive set. For the second category image, we separate them from the negative images to prevent confusion and misdetection. Table 2 summarizes the detailed information about our contribution to SIXray-D.

## 4. Task-Driven Image Cropping by Deep Feature Extraction

**Figure 4:**
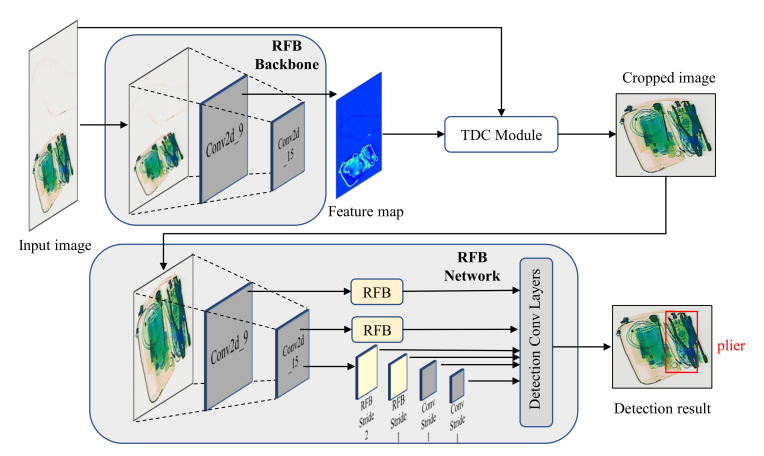
The proposed X-ray image detection pipeline with the TDC module for task-driven cropping. The network backbone for the feature extraction is the same in the detection network. In this work, we use RFB-Net [34] for the detection task, while TDC could also be used for other single-stage detection networks.

**Figure 5:**
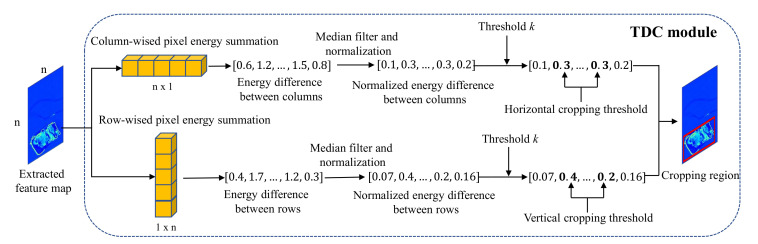
Construction of TDC module by combining the columns/rows wised summation of pixel energy, the median filter to filter out the X-ray artifact spikes, the min-max normalization, and the threshold-based cropping. The cropping region is then combined with the input image to produce the output cropped image.

With the new SIXray-D dataset available, our goal is to develop a more efficient and effective X-ray image detection pipeline that can overcome practical challenges, such as varied image sizes, aspect ratios as well as arbitrary locations of small target objects. We propose the Task-Driven image Cropping (TDC) module, which utilizes activation feature maps from convolutional layers for determining the redundant data to be removed. Figure 4 shows the proposed TDC-based X-ray image detection pipeline, which consists of the TDC module based on the feature map generated by the task network backbone, and the detection process using the output of the cropping module. As shown in Figure 4, the cropping process can utilize the same network backbone with the detection network to save memory and computation. Figure 5 illustrates the detailed structure of the TDC module. Next, we will first introduce preliminaries about CNN feature maps, followed by the detailed TDC module and how it works for adaptive image cropping.

### 4.1. Feature Map Generation

In a CNN model, the feature map $F\in R^{W\times H\times C}$ is the result of the input image after the convolution process, where W and H are the width and height of the feature map, and C is the number of channels. Usually, the first few layers provide details about low-level features such as edges and colors. The deeper layers will provide information about high-level features like positions and shapes of salient objects [54,55].

Let F be the output from a convolutional layer, in which $F=\{F_{1},F_{2},\ldots ,F_{C}\}$ where *C* is the number of channels and $F_{i}$ is the output per channel. To generate an overall feature map of a layer, the sum of magnitudes of each channel output is calculated and then averaged as follows:

(1) $$F_{avg}=\frac{1}{C}\sum_{i=1}^{C}\left|F_{i}\right|,$$

where $|F_{i}|$ is the element-wise absolute value of $F_{i}$ and $F_{avg}$ is also a 2D array.

**Figure 6:**
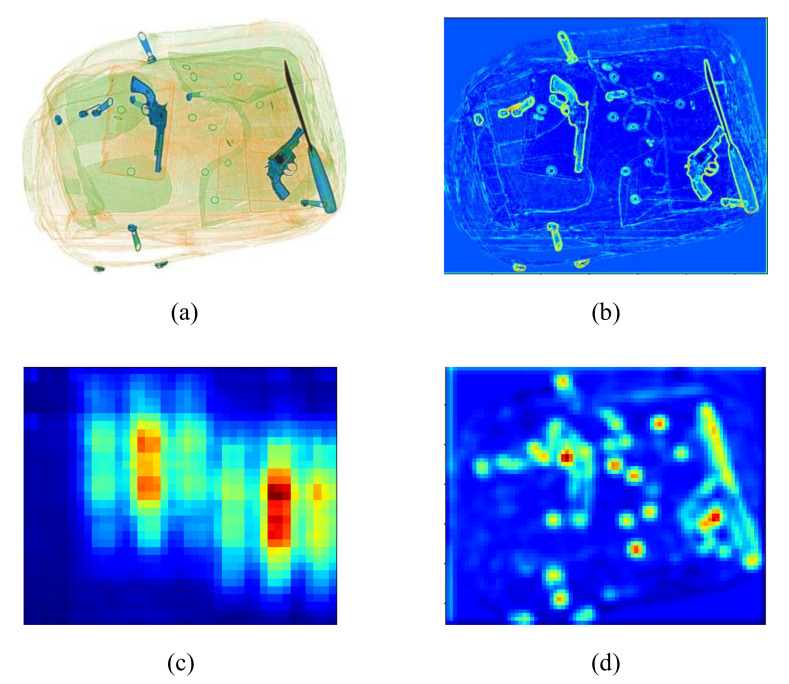
Original X-ray image and feature outputs from different convolutional layers from the backbone of RFB Net. (**a**) is the original image, (**b**) is the 2nd layer output, (**c**) is the 15th layer output and (**d**) is the 9th layer output.

For scanned security X-ray images, objects of interest overlap with the container or luggage. Hence, to provide a suitable feature map that distinguishes the items from the background, we decide to extract middle convolutional layers. Activation from such layer contains moderate information about objects, but does not omit the suitcases containing them, which helps distinguish against the plain background of the X-ray image. Figure 6 presents the original image and three different feature maps from three layers: the 2nd convolutional layer, the 15th convolutional layer, and our choice, the 9th layer of the backbone of RFB Net. Note that we can use the output from any convolutional layer to perform feature extraction. The ablation study in Section 5.4 proves that the 9th layer gives the best detection accuracy improvement after the image cropping process.

### 4.2. TDC Module and Image Cropping

**Dimension reduction of feature matrix.**Figure 5 summarizes our TDC module for efficient task-driven feature-based cropping. We propose that the pixel magnitude of the feature map in the regions of interest is higher in other areas. To prove this idea and utilize it for the cropping process, we construct a column-wised feature matrix $F_{c}$ through summation of all pixels $F_{avg}\left(x,y\right)$ in the feature map $F_{avg}$ vertically:

(2) $$F_{c}=\sum_{j=1}^{H}F_{avg}\left(x,j\right),$$

where *H* is the vertical resolution of the feature map $F_{avg}$. $F_{c}=\left\{F_{c1},F_{c2},\ldots ,F_{cW}\right\}$ where $F_{ci}$ is the magnitude of column *i* in the feature map, and *W* is the horizontal resolution of $F_{avg}$.

**Columns difference and artifact removal.** We take the absolute difference between columns, $F_{diff}$, to observe the rapid change in magnitude between column, and to identify the region of interest as follows:

(3) $$F_{diff}\left(i\right)=\left|F_{c(i+1)}-F_{ci}\right|.$$

**Figure 7:**
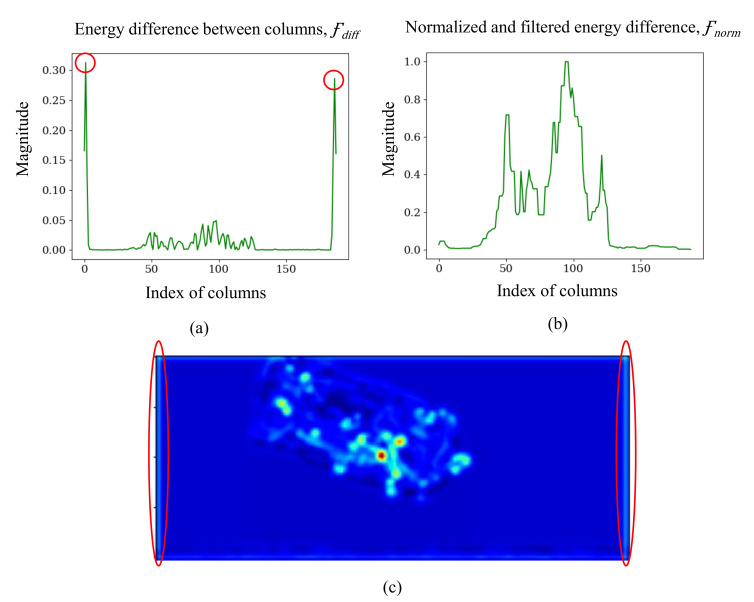
Result of median filtering and normalizing process. (**a**) is the visualization of $F_{diff}$, the energy difference between columns of feature map. (**b**) is the visualization of $F_{norm}$, the result of normalization and filtering process of $F_{diff}$. (**c**) is the feature map, and the spikes labeled by the red circles caused by X-ray artifacts in $F_{diff}$ can be seen in (**a**,**c**), which are removed by the median filter.

However, the vertical and horizontal bars appear as artifacts in the scanned image horizontally and vertically due to the scanner or image processing steps [21]. Such artifacts can cause spikes in $F_{diff}$ shown in Figure 7. We use a median filter to filter our matrix:

(4) $$F_{med}=\Phi \left(F_{diff},n\right),$$

where Φ(x,n) indicates median filter of *x* with kernel *n*. We normalize $F_{diff}$ using min-max normalization to derive $F_{norm}$ as the final column difference matrix:

(5) $$F_{norm}\left(i\right)=\frac{F_{med}\left(i\right)-min\left(F_{med}\right)}{max\left(F_{med}\right)-min\left(F_{med}\right)},$$

where $F_{norm}=\left\{F_{n1},F_{n2},\ldots F_{d(W-1)}\right\}$ indicates the normalized horizontal change in magnitude of the feature map.

**Threshold-based cropping.** We determine a threshold *k* based on $F_{norm}$ to efficiently crop the image without removing salient objects. If $F_{ni}>k$, the rate of change of column *i* is higher than the threshold, and column *i* indicates the start of regions of interest. $F_{norm}$ is scanned from left to right and vice versa to determine the horizontal boundary of the cropping region. We repeat the process vertically to decide the vertical boundary for the region of interest (RoI). After that, the original image will be cropped according to the RoI on the feature map.

## 5. Experiments

### 5.1. Experiment Setup

**Baseline and dataset.** We use some of the most common single-stage detectors with relatively good detection performance, namely SSD [32], RetinaNet [31], and RFB Net [34] to set up the default baseline model for the cropping procedure. The dataset used for benchmarking is the SIXray-D dataset. We follow the PASCAL VOC 2007 dataset structure to split the train-test set. The original SIXray dataset contains 8820 positive images. We randomly split the dataset with a ratio of 90/10 for train/test, and reserve the test set for testing only to provide an unbiased evaluation of the model’s performance. We use the recommended training parameters of SSD-512, RetinaNet, and RFB-512 from publicly available codes in PyTorch [56]. We set the training epochs to 200, and the training takes around 1–2 days to complete for each model.

For TDC, we use the baseline with the best result from the baseline benchmark to perform image cropping. The baseline is used in the image cropping and detection tasks for the test dataset. We use two configurations on the baseline: one is the default configuration, which resizes the input image to 512 × 512, the other is a dynamic input configuration that takes in arbitrary sizes image. We try to use the dynamic input model to increase the detection performance at the cost of model runtime. We use a median filter of kernel 9 to remove the X-ray artifacts and set a baseline threshold *k* of 0.5. We believe a 50% difference between the energy of columns/rows of feature map can indicate the starting of the region of interest, then we tune it to optimize the cropping performance based on two criteria, (1) to achieve the best detection mAP and (2) to prevent over-cropping and cutoff objects of interest. We discover that ranging the threshold *k* from 0.15 to 0.3 achieves the best performance satisfying both criteria, and *k* being 0.15 has the highest detection performance. Thus, *k* is set at 0.15 for both horizontal and vertical cropping.

**Figure 8:**
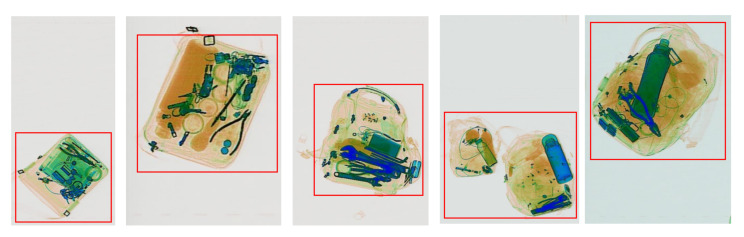
Results from TDC using RFB Net backbone. The red bounding boxes mark the output after cropping when using *k* = 0.15.

To compare the performance of TDC with both conventional and deep learning-based image cropping, we choose two methods: the first is Canny edge detection [40] based cropping implemented using Python 3 [57], and the second method is the aesthetic-based cropping proposed by Peng et al. [24]. For the problem of cropping off white space and preserving the objects of interest, Canny edge detection is a simple and powerful unsupervised method to detect the saliency region. For the deep learning-based cropping, as there is no task-driven based image cropping and almost all of the currently available methods focus on improving the aesthetic aspect of the cropped image, we choose the method that provides the highest aesthetic scores and is available to the public. We follow the recommended parameters and use the pre-trained 512 × 512 model for the aesthetic-based cropping method [24] using TensorFlow [58]. All experiments are conducted on a single NVIDIA GTX 1080Ti GPU. Figure 8 displays some results from TDC where the background of images is cropped off and the main content is preserved.

### 5.2. SIXray-D Benchmarking

In this section, we benchmark the detection performance on the SIXray-D dataset using mean Average Precision (mAP) from PASCAL VOC 2007 metric [59] with Intersection over Union (IoU) of 0.5. Table 3 summarizes the experiment results on the SIXray-D test set with 836 images.

Based on Table 3, RFB Net performs better than RetinaNet and SSD Net. Both SSD and RFB use the same reduced VGG-16 backbone for feature extraction. However, by replacing Conv2D layers in SSD with the RF module to capture features better [34], RFB Net achieves slightly higher mAP than SSD on the test set. Although using a ResNet, which is a deeper backbone [31], RetinaNet cannot match the performance of the above two models. The difference in mAP could come from the difference in architecture and activation function. RetinaNet does not have RF modules and uses FocalLoss (sigmoid activation) [31] while RFB uses Multibox loss (softmax activation) [34]. The softmax activation forces sum of the classification outputs to 1, which is more suitable in multi-classification. Alternatively, sigmoid activation is better for binary classification, hence for a dataset with multiple classes like SIXray-D, softmax in RFB Net is the better loss function. Through the experiment, the RFB Net is the best option for the cropping process baseline.

### 5.3. Cropping Performance Assessment

In this experiment, we further evaluate the effectiveness of the proposed TDC module from two different perspectives: (1) Detection accuracy improvement using a fixed-size RFB Net; and (2) reduction of runtime using dynamic input RFB model when applying TDC on the SIXray-D dataset.

#### 5.3.1. Fixed-Size Model

**Figure 9:**
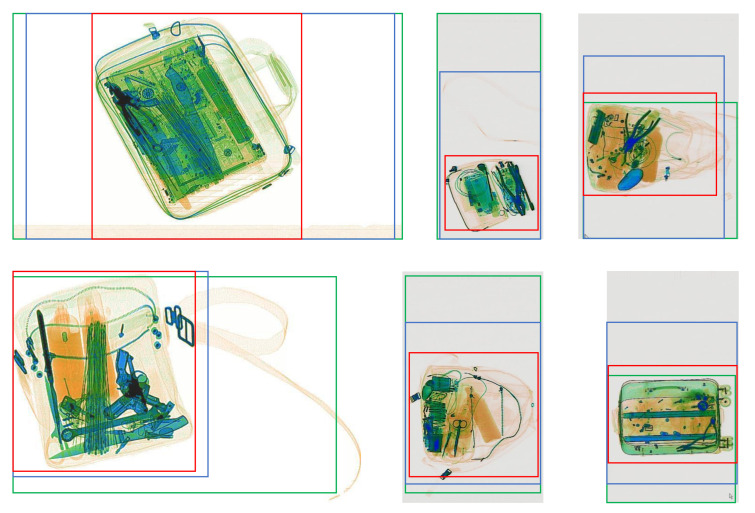
Comparison of cropping output between different methods: Green bounding boxes indicate the outputs from Canny edge [40]-based cropping; Blue bounding boxes indicate the outputs from aesthetic-based cropping [24]; Red bounding boxes indicate the outputs from our proposed TDC module.

For a fixed-size model, it resizes the input images into 512 × 512. It does not retain the objects’ aspect ratios, but the inference time is faster than the dynamic shape input model. Table 3 summarizes the detection performance of RFB net on original and cropped SIXray-D datasets. From Table 3, TDC surpasses other cropping methods and improves the average detection mAP by 2 to the original dataset, which can be credited to the enlargement of small contraband objects such as Scissors and Pliers. While TDC can efficiently crop off the area with X-ray artifacts and redundant objects, the conventional Canny-edge [40] based cropping and deep learning-based aesthetic cropping [24] struggle to do so. Figure 9 illustrates several outputs of the three cropping methods on the SIXray-D dataset. An observation can be made that only TDC can detect the X-ray bar artifacts, the straps of the bags, and the mouse cursors on the image as noise, while the other two methods fail to recognize them, hence leading to less efficient cropping and less space reduced.

#### 5.3.2. Dynamic Shape Input Model

As the RFB Net reuses the architecture of SSD, anchor boxes are introduced in the convolutional layers [32]. While these boxes are static in the original network, the anchor boxes are re-calculated for every single image in the dynamic shape model. Hence, this will drastically increase the runtime of the detection model. When using dynamic input shape, our goal is to achieve a better detection mAP at the cost of slower runtime. By reducing the input size through feature-based cropping, the time for anchors generation will decrease, and subsequently, the overall inference time will reduce. Table 4 summarizes the detection result when applying different cropping methods on the SIXray-D dataset and using dynamic input shape RFB Net. In this experiment, TDC proves to be the best cropping method with the highest mAP and lowest runtime compared to the other methods. The average inference time on the Dynamic RFB + TDC is decreased by 8% compared to the original set. The overall mAP is almost the same across all the cropping methods, with TDC being 0.3 mAP higher. This can be credited to the removal of the redundant background area, in which TDC surpasses other approaches as shown in Figure 9.

### 5.4. Ablation Study

**Figure 10:**
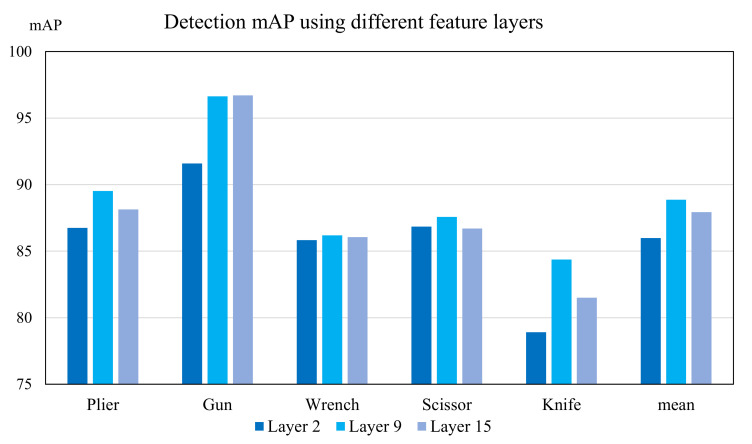
Ablation study: detection AP over each class (and the mean) of SIXray-D dataset using the RFB + TDC detection pipeline. The feature outputs are varied from the layer 2 to 15 of the RFB Net, which are used to crop the X-ray images using TDC, thus generating different detection results.

In this section, we assess the effect on detection accuracy using features extracted from different convolutional layers in the backbone VGG model of the RFB Net and summarize the result in Figure 10. We use the 2nd, 9th and 15th layers to represent the low, medium, and deep feature layers’ outputs. An observation can be made that using the features from the 9th convolutional layer for the TDC module gives the best detection result. The lower layers only highlight contraband objects and make the algorithm cut off the important background and potential objects of interest. Meanwhile, the deep layers’ features provide little change between columns and rows of the image as the whole baggage is highlighted, thus making the cropping algorithm ineffective. By using the middle convolutional layer, we can preserve the crucial background and cut off the unwanted redundant spaces in the process.

## 6. Conclusions

In this work, we attempted to tackle the practical challenges in X-ray image detection tasks. We proposed a fully-annotated SIXray-D dataset with completed positive samples with annotation boxes to benchmark the X-ray image detection tasks. We proposed the TDC module, a novel task-driven image cropping method that can effectively improve the X-ray image detection pipeline. We conducted extensive experiments showing that our proposed TDC-based X-ray detection scheme can reduce the run time for the dynamic shape input RFB net and increase the mAP for the fixed-size counterpart. The proposed TDC scheme is simply based on feature extraction without additional assumptions on the specific detector, thus it can be extended to other single-stage deep detection models as well. We plan to further investigate the effect of maintaining salient objects’ aspect ratios and apply the loss to control the threshold of the cropping method in future work.

## Figures and Tables

**Table 1:** Key attributes of popular detection security X-ray datasets, namely GDXray [14] and OPIXray [13], as well as the proposed SIXray-D dataset.

Dataset	Class Types	Positive Images	Negative Images	Multiple Objects per Image	Object Occlusion	Real X-ray Artifacts	Realistic Orientation of Luggage
GDXray	Shuriken, gun, knife	8850	10,550	✗	✗	✗	✗
OPIXray	Scissors and variants of knife	8885	0	✗ ${}^{1}$	✓	✗	✗
SIXray-D	Scissors, pliers, gun, wrench, knife	11,401	1,050,302	✓	✓	✓	✓

^1^ For OPIXray, there are only 35 out of 8885 images with multiple objects per image.

**Table 2:** Comparison between SIXray [17] and SIXray-D datasets.

Dataset	SIXray	SIXray-D
Supervised task	Classification	Detection
Bounding box annotations	Test Set	Train + test set
Positive images	8823	11,401
Positive objects	20,729	23,470

**Table 3:** Detection Average Precision on each class and mean Average Precision (mAP) on different models and test datasets. **Red** indicates the best and *blue* indicates the second best performance.

Method	Pliers	Gun	Wrench	Scissors	Knife	Mean
SSD	87.03	96.31	84.73	84.04	82.51	86.92
RetinaNet	82.73	84.51	75.69	79.95	74.64	81.50
RFB	88.78	96.13	85.92	84.73	83.22	87.76
RFB + Edge [40] based crop	88.79	95.85	86.12	*86.04*	*83.93*	88.16
RFB + Aesthetic crop [24]	*89.43*	*96.32*	*86.17*	85.48	83.43	*88.38*
RFB + TDC	**89.52**	**96.63**	**86.19**	**87.57**	**84.37**	**88.86**

**Table 4:** Detector performance on SIXray-D datasets using dynamic shape input RFB Net (Dynamic RFB) after applying different cropping methods. **Red** indicates the best results and *blue* indicates the second best results.

Method	Pliers	Gun	Wrench	Scissors	Knife	Mean	Runtime (s) ↓	Runtime Reduction (%) ↑
Dynamic RFB	*90.83*	**98.67**	87.26	91.65	83.01	90.28	2.394	N/A
Dynamic RFB + Canny edge [40]-based crop	89.84	97.93	88.20	90.80	**84.69**	90.29	2.271	5.13
Dynamic RFB + Aesthetic crop [24]	90.52	98.36	**88.76**	89.31	*83.90*	*90.37*	*2.221*	*7.23*
Dynamic RFB + TDC	**91.07**	*98.54*	*88.51*	**92.32**	82.78	**90.60**	**2.192**	**8.44**

## Data Availability

The publicly available SIXray-D dataset was analyzed in this study. This data can be found here: https://www.ntu.edu.sg/rose/research-focus/datasets (accessed on 13 August 2021) Note that we only contribute the extra annotations for the detection task. The images are all from the existing SIXray dataset (https://github.com/MeioJane/SIXray) (accessed on 13 August 2021).
